# Dose-Dependent Effects of Oral Tyrosine Administration on Plasma Tyrosine Levels and Cognition in Aging

**DOI:** 10.3390/nu9121279

**Published:** 2017-11-23

**Authors:** Ondine van de Rest, Mirjam Bloemendaal, Rianne de Heus, Esther Aarts

**Affiliations:** 1 Wageningen University, Division of Human Nutrition, P.O. Box 8129, 6700 EV Wageningen, The Netherlands; Rianne.deHeus@radboudumc.nl; 2 Radboud University, Donders Institute for Brain, Cognition and Behaviour, Centre for Cognitive Neuroimaging, P.O. Box 9101, 6500 HB Nijmegen, The Netherlands; mirjambloemendaal@gmail.com (M.B.); e.aarts@donders.ru.nl (E.A.); 3 Radboud University Medical Center, Donders Institute for Brain, Cognition and Behaviour, Department of Geriatric Medicine, P.O. Box 9101, 6500 HB Nijmegen, The Netherlands; 4 Radboud Alzheimer Centre, P.O. Box 9101, 6500 HB Nijmegen, The Netherlands

**Keywords:** tyrosine, dose-response, aging, working memory, plasma amino acids, catecholamines, dopamine

## Abstract

The effects of tyrosine on plasma response and cognition in aging are unknown. We assessed the dose-dependent response to tyrosine administration in older adults in both plasma tyrosine concentrations and working memory performance. In this double blind randomized cross-over trial 17 older adults (aged 60–75 years) received a single administration of 100, 150, or 200 mg/kg body weight of tyrosine. For comparison, 17 young adults (aged 18–35 years) received a dose of 150 mg/kg body weight of tyrosine. Tyrosine plasma concentrations were determined before and 90, 120, 150, 180, 210, and 240 min after tyrosine intake. Working memory was assessed using the N-back task at 90 min after tyrosine administration. Older adults showed a dose-dependent increase in plasma tyrosine concentrations (*p* < 0.001), and the plasma response was higher than for young adults with the same dose (*p* < 0.001). Load-dependent working memory performance decreased with higher doses of tyrosine (*p* = 0.048), especially in older adults with greater dose-dependent plasma tyrosine responses (*p* = 0.035). Our results show an age-related increase in plasma tyrosine response, which was associated with a dose-dependent decline in cognitive functioning in older adults.

## 1. Introduction

Tyrosine is one of the conditionally essential amino acids and is particularly found in protein-rich foods such as dairy, meat, fish, eggs, seeds, nuts, and beans. It can also be synthesized from the essential amino acid phenylalanine, but this is dependent on sufficient availability of this precursor [1].

Studies of tyrosine supplementation in young adults showed that after doses of 100 mg/kg body weight and 150 mg/kg body weight, peak plasma concentrations of the compound were reached approximately two hours after ingestion. The increase in plasma tyrosine concentrations was more pronounced and longer persisting after the 150 mg/kg body weight dose than after the 100 mg/kg body weight dose [2]. With aging, changes in peripheral tyrosine absorption and metabolism, for example in liver [3], muscles [4], and melanocytes [5], may occur and, consequently, the same dose of tyrosine may lead to a different response in plasma (i.e., peripheral) tyrosine concentrations in older relative to young adults. We aimed to investigate this in older adults with three tyrosine doses using a randomized cross-over design (100 mg/kg, 150 mg/kg, and 200 mg/kg body weight of tyrosine) and a 150 mg/kg body weight dose of tyrosine in young adults for comparison.

Tyrosine is the precursor of the neuromodulatory catecholamines dopamine and noradrenaline [6,7]. In animal models, concentrations of brain tyrosine were shown to be modifiable by dietary intake [8] and tyrosine administration enhanced central catecholamine synthesis in rodents [9,10,11,12] and in humans [13]. Catecholamines, including dopamine, are important for working memory [14,15]. Several studies in young adults have shown that tyrosine administration can reverse working memory impairments under stressful conditions (for reviews, see [16,17]), or increase cognition, including working memory, in regular environmental conditions [18,19,20].

Aging is characterized by a decline in brain dopamine receptor and transporter binding [21], which is accompanied by impairments in working memory [22,23,24]. Therefore, older adults may also cognitively benefit from tyrosine supplementation, perhaps with increasing dose. Alternatively, increasing tyrosine doses could potentially hamper cognition, as aging has also been shown to be accompanied by (compensatory) up-regulation of dopamine synthesis capacity [25,26], which has been related to, if anything, worse, rather than better, neurocognitive functioning relative to young adults [25,27]. Moreover, it is unknown how age-related peripheral effects of tyrosine administration would affect central (i.e., cognitive) functioning. Therefore, in addition to our primary aim into the effects of tyrosine administration on plasma response, we explored the effects of the three tyrosine doses on working memory performance in older adults and its relation to the plasma response. In the absence of a placebo condition, we can only study dose-dependent effects of tyrosine administration. Hence, our main secondary aim was to assess whether working memory performance would increase or decrease with increasing tyrosine dose in older adults.

## 2. Materials and Methods

### 2.1. Participants

We included 17 young (17–35 years of age) and 17 older adults (60–75 years of age) with a body mass index (BMI) between 18.5 and 27 kg/m^2^, who were Dutch-speaking and had a normal or corrected-to-normal vision. Participants were recruited through an existing database of volunteers with interest in participating in studies at Wageningen University. Exclusion criteria were: (1) smoking; (2) thyroid problems; (3) use of tyrosine supplements or medication that can interfere with tyrosine’s action; (4) following a low-protein diet as prescribed by a dietician or physician; (5) alcohol consumption >14 (women) or >21 (men) units per week; (6) Mini-Mental State Examination (MMSE) score <24 (only for older adults) [28]; (7) Intelligence Quotient (IQ) < 85 as estimated by the Dutch version of the National Adult Reading Test (NART) [29]; (8) intestinal problems that affect nutrient absorption; (9) Parkinson’s Disease, history of depression or other clinically-significant psychiatric or neurological disorder; (10) under treatment for cardiac or vascular diseases and use of medication for these conditions; (11) being allergic or having a dislike for the product carrier (banana-flavoured yoghurt); (12) bad venous access as judged by the research nurse; and (13) general medical conditions affecting test performance, such as repetitive strain injury (RSI) or sensorimotor handicaps, as judged by the investigator. In addition, performance on the N-back task during the screening test session (15 min-session without feedback) needed to be >60% on levels 0-, 1- and 2-back to assure participants performed according to the instructions. If performance after two practice sessions with feedback and a 15-min test session without feedback was <60%, participants performed one more practice session with feedback and 15-min-session without feedback. If performance was still <60%, participants were excluded. The performance score was calculated by hits/(hits + misses + incorrect rejections + false alarms) × 100% for each level. The Wageningen University Medical Ethical Committee (METC-WU) approved the study on 6 October 2014 (protocol number 14/20). Participants gave written informed consent and were compensated for participation. This trial was registered at www.trialregister.nl as NTR4846.

### 2.2. Study Design

Participants were screened between October 2014 and November 2014, and intervention took place between November and December 2014. A pre-screening comprising an initial check on eligibility criteria was performed by phone. When potential participants fulfilled to the criteria, they were invited for a screening visit during which they were informed about study details and signed the informed consent after which possible inclusion based on in- and exclusion criteria (see Participants) was further determined. Furthermore, participants were weighed and familiarized with the N-back task, which was repeated during the test visits.

**Figure 1:**
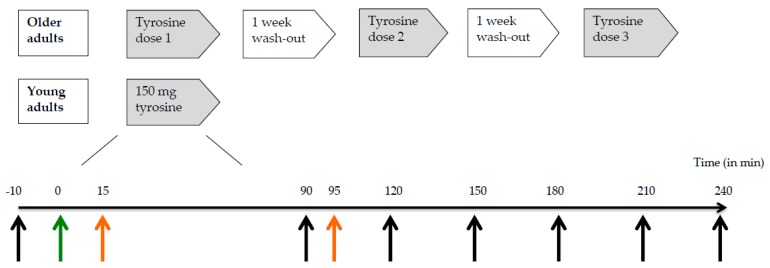
Study design.

We used a double-blind, randomized, cross-over design to investigate the response in plasma tyrosine concentrations following administration of a single dose of 100, 150, or 200 mg/kg body weight of tyrosine to healthy older adults (Figure 1). There was at least one week between the different test conditions (i.e., doses). A reference group of young adults received a dose of 150 mg/kg body weight tyrosine. Test sessions took place in the morning after an overnight fast (10–12 h). An independent researcher randomized the older adults over the three different doses of tyrosine by means of six computer-generated counterbalanced orders.

Older adults (*n* = 17) ingested 100, 150 or 200 mg/kg body weight of tyrosine on three test days in a random order with one week in between. Young participants (*n* = 17) performed only one test day on which they received a dose of 150 mg/kg body weight of tyrosine. The timeline below represents a test day in minutes. Black arrows: time points of blood collection for plasma tyrosine concentrations, green arrow: time point of tyrosine supplementation, and orange arrows: time points of start of practicing (*t* = 15) and performance of N-back working memory task (*t* = 95).

### 2.3. Tyrosine Supplementation

The doses of L-tyrosine powder (Bulkpowders™, Sports Supplements Ltd. Colchester, Essex, United Kingdom) were mixed with banana-flavored yoghurt (Arla^®^ Foods Nederland, Nijkerk, The Netherlands) in a 1:20 ratio to ensure comfortable ingestion of the product. Weighing of the doses and preparing and coding the samples were performed by a staff member not involved in the study. Participants had to consume the whole portion within 10 min, together with one glass of water (150 mL).

### 2.4. Sample Size Calculation

Sample size calculation was based on plasma tyrosine concentration, the primary study parameter. We calculated the effect size of a comparable previous study that assessed plasma tyrosine concentrations in young adults after doses of 100 and 150 mg/kg body weight of tyrosine [2]. Given the post-hoc calculated effect size in this study (*d* = 0.88), the a priori sample size calculation for the current study indicated a sample of 13 participants, using an alpha of 0.05 and a beta of 0.8. Sample size for the secondary objective, i.e., differences in working memory performance for the three tyrosine doses in older adults, was calculated based on the effect size of a study that tested the effect of tyrosine supplementation on N-back performance in young adults [18]. Calculations with this effect size of *d* = 0.7, resulted in a sample size of 17 participants. To have sufficient power for both the primary and the secondary objective we included 17 participants per group.

### 2.5. Cognitive Performance: N-back Working Memory Task

**Figure 2:**
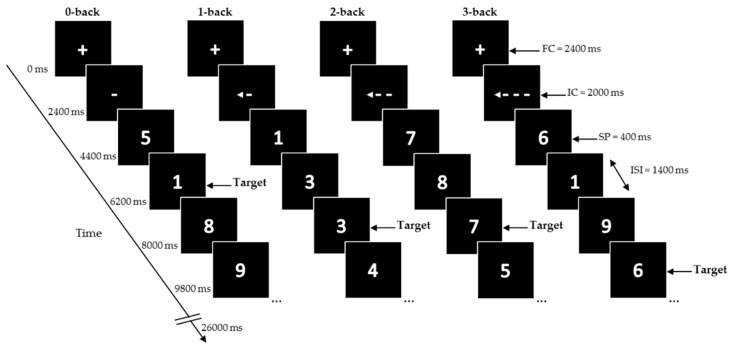
Schematic overview of the numerical N-back task.

Participants performed four conditions of a numerical (digits 0–9) N-back task on the computer (previously described in [30,31] (Figure 2)): a control condition, where a single digit is specified as the target (0-back) and conditions with increasing cognitive load, where the target is any digit identical to the digit presented *n* trials prior (1-back, 2-back, and 3-back). The digits were presented in white in the center of a black screen using a blocked design with 32 blocks (eight blocks for each condition).

At the start of the task, and in between blocks, a fixation cross (FC) was presented for 2400 ms. Each block started with a presentation of the instruction cue (IC) for 2000 ms, followed by 12 trials of single digits. Stimulus presentation (SP) was 400 ms, inter-stimulus interval (ISI) 1400 ms. Total duration of one block was 26,000 ms.

At the start of the task and in between blocks, a fixation cross was presented for 2400 ms. Each block started with the presentation of an instruction cue (IC) for 2000 ms, followed by 12 trials of single digits. During the trials the IC was constantly displayed on the screen. Each digit was presented for 400 ms and followed by an inter-stimulus interval (ISI) of 1400 ms. Total duration of one block was 26,000 ms. Blocks were presented in a mirrored design. Each block contained a pseudorandom sequence with either two or three targets, or no more than two consecutive targets, making a total of 20 targets within each condition. A different sequence was used for each test session. The total task duration was 14 min. Participants pressed the left mouse button using the right-index finger for targets. During the test visit, the task was practiced once more with and without feedback, right after consuming the study product. The actual N-back task was performed 90 min after tyrosine consumption, the moment that we expected the tyrosine levels to begin peaking, as based on previous studies [2,32]. The task was presented on a laptop using Presentation 17.1 software (Neurobehavioural Systems, Inc., San Francisco, CA, USA). A standard protocol was used, and all tests were performed in the same quiet room.

### 2.6. Plasma Tyrosine Concentrations

Plasma tyrosine concentrations were assessed at seven time points, i.e., before tyrosine consumption (T0), and 90, 120, 150, 180, 210, and 240 min after tyrosine consumption. A peripheral venous catheter was used to collect the blood samples into 10 mL ethylenediaminetetraacetic acid (EDTA) vacutainers. The catheter was flushed with a solution of 0.9% of NaCl to keep it open. Directly after collection, blood samples were centrifuged during 10 min at 1200× *g* and a temperature of 4 °C, and plasma was transferred to 1 mL microvial tubes and stored at −80 °C until laboratory analyses. Plasma tyrosine concentrations were quantified by hydrophilic interaction liquid chromatography (HILIC) coupled with tandem mass-spectrometry as described by Prinsen and colleagues [33].

### 2.7. Other Measurements

We obtained information on educational level by questionnaire and education was categorized according to Statistics Netherlands (CBS). Body height was measured at baseline with a wall-mounted stadiometer to the nearest 0.1 cm. Body weight was measured to the nearest 0.1 kg with a calibrated digital scale at the screening visit, with participants dressed in underwear. There was a maximum of four weeks between the screening visit and the first test session and we instructed participants not to change their diet, activity pattern and lifestyle during the study, so that the weight measured during the screening visit was a reliable measure of someone’s weight at the test session. At the start of each test session adherence to this instruction was checked with the participant.

### 2.8. Statistical Analysis

Data analyses were performed using IBM SPSS Statistics 22.0. Differences between older and young adults on demographic values (age, sex, education, estimated verbal IQ, body weight, BMI, MMSE) were determined using two-sample t-tests or chi-square tests. Plasma tyrosine concentrations following tyrosine administration are visualized over time using mean and standard error of the mean (SEM) per dose and age group. Outliers were determined based on Grubbs test [34]. Baseline differences in plasma tyrosine concentrations between young and older adults were determined using a one-way Analysis of Variance (ANOVA) on tyrosine levels after 150 mg/kg body weight at T0, with age group (young, older adults) as the grouping variable. Baseline differences between the three dosages within the older adult group were assessed using repeated measures (RM) ANOVA with factor Dose (T0 tyrosine levels at baseline for doses 100, 150 mg/kg, and 200 mg/kg body weight). The effect of age on plasma tyrosine concentrations after 150 mg/kg body weight tyrosine administration, at baseline corrected time points (values of all time points minus T0), was assessed using RM ANOVA with within-subjects factor Time (T90minT0, T120minT0, T150minT0, T180minT0, T210minT0, and T240minT0) and between-subjects factor Age (young, older adults). Differential effects of the three dosages on plasma tyrosine concentrations within older adults were determined using RM ANOVA with within-subjects factors Dose (100, 150, and 200 mg/kg) and Time (T90minT0, T120minT0, T150minT0, T180minT0, T210minT0, and T240minT0).

Tyrosine effects on working memory (i.e., N-back) performance was only assessed for the older adults, as their performance could be compared between three doses. We also compared N-back performance between the two age groups at the same dose (i.e., 150 mg/kg body weight) to replicate the well-known age-related impairments in working memory (see Introduction). Percentage hits, misses, correct rejections and false alarms were calculated as well as reaction times (RT) for hits and false alarms. Within-subject RM ANOVAs using factors Dose (100, 150, and 200 mg/kg body weight) and Cognitive Load (0-back, 1-back, 2-back, and 3-back) were used to compare percentages of hits and false alarms as well as RT of hits on the N-back task among the three tyrosine doses. Furthermore, we tested for an effect of individual differences in dose-related plasma level increase on N-back performance (percentage of hits and false alarms and RTs). For this purpose, the slope of plasma level increase as a function of dose was calculated per subject. This was done by subtracting the baseline (T0) from the peak measurement (T90) per dose and calculating the slope as a function of the three increasing doses, resulting in one beta value per subject. Subsequently, ANOVAs were performed using the within-subject factors Dose (100, 150, and 200 mg/kg body weight) and Cognitive Load (0-back, 1-back, 2-back, and 3-back) and between-subject factor Plasma level increase (median split of slope (beta) values into low and high tyrosine-induced plasma level increase).

Upon significant results, simple effects were assessed. We considered a two-sided *p*-value < 0.05 as significant.

## 3. Results

### 3.1. Participants

**Figure 3:**
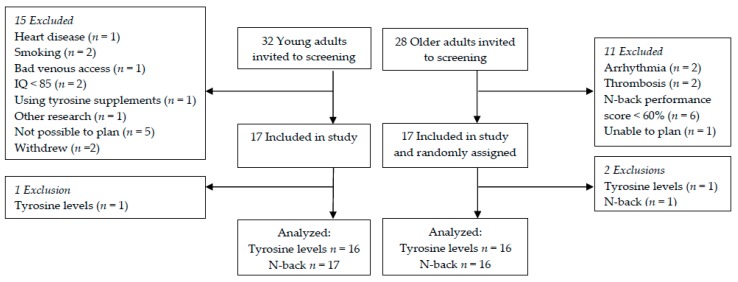
Flowchart of participants through the study. Abbreviation: IQ, Intelligence Quotient.

The flowchart of participants through the study is shown in Figure 3. For data analyses of plasma tyrosine concentrations, one young adult was excluded based on extremely high plasma tyrosine concentrations at T90, T180, and T240 and one older adult because of extremely low plasma tyrosine concentrations at all time points after 100 mg/kg body weight, T150, T210, and T240 after 150 mg/kg body weight and all time points but T0 after 200 mg/kg body weight. N-back data after the 150 mg/kg body weight dose were missing for one older adult, who was subsequently excluded from the analyses including this dose. Therefore, N-back data analyses within the older adults including this dose are based on 16 individuals instead of 17. For baseline characteristics of the older and young adults, see Table 1. 

Age groups were no different in sex and body weight; the latter implicates that the absolute tyrosine dose also did not differ between the age groups. Older adults received less education, but displayed a higher estimated IQ (adjusted Dutch NART score) relative to young adults. BMI was higher for older than for young adults, but did not interact with our plasma outcome measures when added as covariate in the analyses (all *p*-values > 0.43). Tyrosine administrations were well tolerated; no adverse events were reported.

### 3.2. Age- and Dose-Dependent Tyrosine Effects on Plasma Tyrosine

#### 3.2.1. Age and Time Course Comparison at 150 mg/kg Body Weight Dose

**Figure 4:**
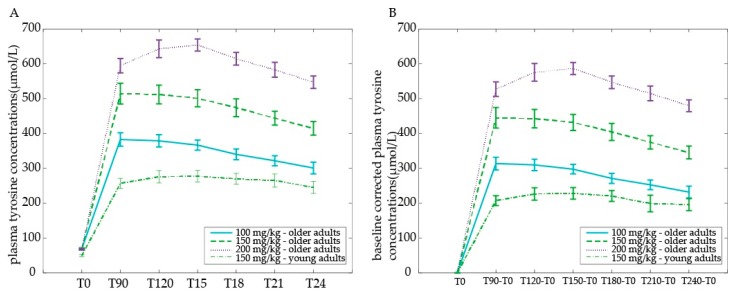
Plasma tyrosine concentrations after tyrosine administration, by tyrosine dose and age group.

At baseline, tyrosine levels were higher for older compared with young adults (main effect of Age group: *F*(1,30) = 23.35, *p* < 0.001) (Figure 4a). When corrected for baseline differences (all time points minus T0), older adults displayed a higher increase in plasma tyrosine concentrations than young adults across time points (main effect of Age group *F*(1,30) = 55.17, *p* < 0.001), driven by all baseline-corrected time points (T90minT0: *F*(1,30) = 53.15; T120minT0: *F*(1,30) = 45.51; T150minT0: *F*(1,30) = 51.20; T180minT0: *F*(1,30) = 40.10; T210minT0: *F*(1,30) = 33.45; T240minT0: *F*(1,30) = 35.25; all *p* < 0.001) (Figure 4b). Moreover, tyrosine levels followed a different time course in older compared with young adults (Time by Age group interaction: *F*(5,150) = 4.11, *p* = 0.002). Young adults did not show a significant decrease with time (*F*(1,15) = 2.02, *p* = 0.086), but plasma levels decreased in the older adults group after T150 onwards (main effect of time: *F*(1,15) = 11.70, *p* < 0.001).

In sum, we observed higher baseline plasma tyrosine concentrations in older compared with young adults. Moreover, after receiving the same dose of a 150 mg/kg body weight, older adults displayed a higher (baseline-corrected) plasma level increase than young adults.

Plasma tyrosine concentrations are given as μmol/L (mean ± SEM) 0, 90, 120, 150, 180, 210, and 240 min after tyrosine administration: (a) raw values plotted for illustration purposes; (b) baseline-corrected values used for statistical analyses by comparing dose response curves.

#### 3.2.2. Dose Comparisons within Older Adults

Within the older adults group, baseline tyrosine levels at T0 did not differ among dosages. We observed a clear dose-dependent response after administration of 100, 150, or 200 mg/kg body weight of tyrosine in the older adults (Figure 4a). Specifically, a higher dose resulted in higher tyrosine plasma levels relative to a lower dose (main effect of Dose on baseline corrected tyrosine levels: *F*(2,30) = 151.18, *p* < 0.001), significant between all three doses (100 vs. 150 mg/kg body weight: *F*(2,30) = 48.35, *p* < 0.001; 100 vs. 200 mg/kg body weight: *F*(1,15) = 361.32, *p* < 0.001; 150 vs. 200 mg/kg body weight: *F*(2,30) = 121.32, *p* < 0.001). Moreover, the time course of plasma levels was different between doses 200 mg/kg and 100 mg/kg and 150 mg/kg body weight (Time (6) × Dose (100, 150, 200) interaction: *F*(10,150) = 3.30, *p* = 0.001, driven by a Time (6) × Dose (100, 200) interaction: *F*(5,75) = 4.80, *p* = 0.001 and a Time (6) × Dose (150, 200) interaction: *F*(5,75) = 4.30, *p* = 0.002 instead of a Time (6) × Dose (100, 150) interaction: *F*(5,75) < 1). After the 200 mg/kg body weight dose, plasma levels increased further after T90 peaking at T120 compared with the 100 mg/kg body weight (Time (T90, T120) × Dose (100, 200) interaction: *F*(1,15) = 9.32, *p* = 0.008) and compared with the 150 mg/kg body weight (Time (T90, T120) × Dose (150, 200) interaction: *F*(1,15) = 9.09, *p* = 0.009). Furthermore, across doses, baseline-corrected tyrosine levels changed as a function of time (main effect of Time: *F*(5,75) = 13.80, *p* < 0.001), with plasma levels decreasing after T150 onwards (Figure 4b).

In sum, tyrosine plasma levels in older adults increased with increasing dose. The peak was reached half an hour later after the highest compared with the lower doses: plasma levels peaked at 90 min after the 100 mg/kg and 150 mg/kg body weight dose, but at 120 min after the 200 mg/kg dose in older adults. Following all doses, tyrosine levels decreased from 2.5 h after ingestion, not returning to baseline levels within four hours.

### 3.3. Dose-Dependent Tyrosine Effects on Working Memory Performance

For accuracy scores and RTs, see Table 2. Total percentage of hits (across levels) was greater than 60%, i.e., above chance.

As expected, RT slowed down, hits decreased and false alarms increased as a function of N-back level difficulty across age groups (main effect of Cognitive load, hits: *F*(3,45) = 70.28, *p* < 0.001; false alarms: *F*(3,45) = 25.58, *p* < 0.001; RT: *F*(3,45) = 72.33, *p* < 0.001), and older adults performed worse on the N-back task than young adults on the same dose of tyrosine (i.e., 150 mg/kg body weight; hits: *F*(3,93) = 83.83, *p* < 0.001; false alarms: *F*(3,93) = 29.82, *p* < 0.001; RT: *F*(3,93) = 57.04, *p* < 0.001).

**Figure 5:**
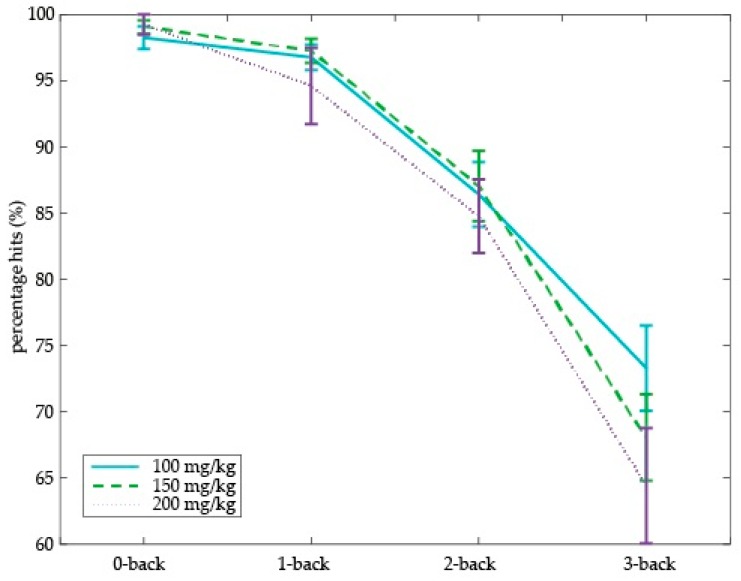
Effect of tyrosine dose on hits in the N-back task as a function of working memory load in older adults. Accuracy is in terms of percentage hits with Standard Error of the Mean (SEM).

Next, we assessed the effect of different doses of tyrosine on working memory performance (i.e., percentage of hits) in older adults. We found a dose-dependent tyrosine effect on working memory in older adults (Dose × Cognitive load (*F*(6,90) = 2.23, *p* = 0.048) (Figure 5). This dose-dependent effect was driven by performance on the highest working memory load, i.e., on the 3-back level (main effect of Dose: *F*(2,30) = 3.38, *p* = 0.047). Specifically, relative to the 100 mg/kg body weight dose, 3-back performance decreased on the 150 and 200 mg/kg dose (150 vs. 100 mg/kg: *F*(1,15) = 4.98, *p* = 0.041; 200 vs. 100 mg/kg: *F*(1,16) = 6.89, *p* = 0.018). We did not observe performance differences on the 3-back level between 150 and 200 mg/kg body weight. On the other working memory loads (i.e., 0-, 1-, and 2-back), no main effect of dose was observed. No main effect of dose or interactions with cognitive load were observed on percentage of false alarms or RTs.

**Figure 6:**
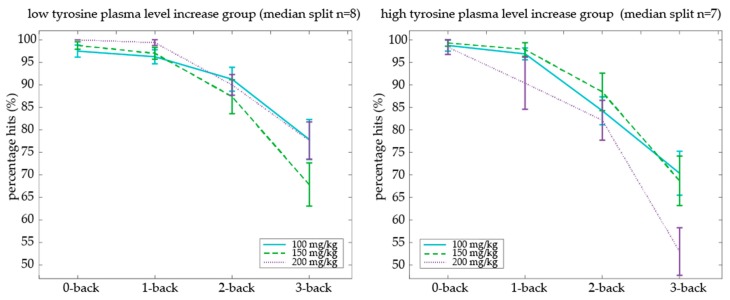
Effect of tyrosine dose and cognitive load on accuracy in older adults who had either a low or high dose-dependent plasma tyrosine increase (based on a median split).

To relate plasma increase of tyrosine to working memory performance, we calculated individual beta (i.e., slope) values of tyrosine plasma increase (T90-T0) as a function of increasing oral dose. Participants with a lower versus higher dose-dependent plasma level increase were separated by a median split on their plasma increase beta values (range plasma level increase: 50.85–182.8, median split on beta of 105.5, low increase group *n* = 8, high increase group *n* = 7). We used an ANOVA with factors Dose (100, 150, and 200 mg/kg body weight) × Cognitive Load (0-back, 1-back, 2-back, 3-back) × Plasma level increase groups (low versus high beta values of dose-dependent plasma level increase) to assess the effect of individual differences in plasma level increase on N-back performance. We observed that the above-mentioned dose-dependent tyrosine effects on N-back performance were indeed dependent on whether the older adult had a low or high dose-dependent increase in tyrosine plasma values (Dose × Cognitive Load × Plasma increase group: *F*(6,78) = 2.40, *p* = 0.035) (Figure 6). This effect was driven by the high plasma increase group, showing a Dose × Cognitive Load interaction (*F*(6,36) = 2.85, *p* = 0.023), not the low plasma increase group (*F*(6,42) = 1.78, *p* = 0.123). Specifically, within the high plasma increase group, the percentage of hits decreased with increasing cognitive load in the 200 mg/kg body weight dose compared with 100 mg/kg body weight (Dose × Cognitive Load interaction: (*F*(3,21) = 5.32, *p* = 0.01). Percentage of hits as a function of cognitive load was not different between 150 and 100 or 200 mg/kg body weight doses (*F*(3,18) = 1.88, *p* = 0.168 and *F*(3,18) = 1.51, *p* = 0.245, respectively).

Percentage of hits was also differentially modulated by the Dose and Plasma increase group independent of Cognitive Load (dose × plasma increase group interaction: (*F*(2,26) = 6.25, *p* = 0.006). In the high-plasma increase group, we observed a trend towards lower accuracy overall as a function of dose, whereas in the low-plasma increase group we observed a trend in the other direction (main Dose: *F*(2,14) = 3.54, *p* = 0.057 and *F*(2,12) = 3.61, *p* = 0.059, for the high and low plasma increase group respectively). No interactions between Dose or Cognitive Load with the Plasma increase group were observed on percentage false alarms or RTs.

In sum, relative to the lowest dose, higher tyrosine doses resulted in a lower percentage of hits on the highest working memory load (i.e., 3-back level) in older adults. This adverse dose-dependent tyrosine effect on working memory performance was particularly evident in older adults who also had higher dose-dependent increases in plasma tyrosine concentrations, providing a link between our psychological and physiological findings.

## 4. Discussion

To the best of our knowledge this is the first study investigating the effects of tyrosine administration on its plasma concentrations and cognitive effects in older adults. Using a cross-over design, our findings showed a clear dose-related increase in plasma tyrosine concentrations after administration of 100, 150, or 200 mg/kg body weight of tyrosine. Importantly, we observed that older adults had a higher tyrosine plasma levels than a comparison group of young participants after the same dose of 150 mg/kg body weight tyrosine. Furthermore, this study demonstrates unfavorable effects of higher doses (i.e., 150 and 200 mg/kg body weight) of tyrosine relative to a lower dose (i.e., 100 mg/kg body weight) on working memory performance in older adults. This dose-dependent decline in terms of N-back hits was especially seen in older adults with higher dose-dependent increases in plasma tyrosine concentrations.

Baseline plasma tyrosine concentrations of the young adults were significantly lower compared with those of the older adults, as observed previously in women [35]. Therefore, we adjusted the dose-response analyses for baseline tyrosine levels. Our time courses for tyrosine were similar to those in the previous studies using a 150 mg/kg body weight dose [2,32]. However, where the increase in tyrosine levels was two- to three-fold in those studies, the increase in our study was five- to seven-fold in both young and older adults after the same doses of tyrosine. The larger increase in our study could be caused by the fact that previous studies have been performed with semi-quantitative and, therefore, less precise analysis methods for plasma tyrosine concentrations. Another reason might be the type of carrier used for oral ingestion. The previous studies mixed the tyrosine either in water [2] or apple sauce [32], whereas we used banana-flavored yoghurt to ensure comfortable ingestion (see also [36]). Yoghurt, however, contains amino acids by itself, as well as tyrosine and other large neutral amino acids (LNAAs). However, since our tyrosine doses were multiple times higher (i.e., the amount of tyrosine in the yoghurt was only 4% of the amount we supplied) and, thus, prevailing, we do not expect this has affected our results in comparison with other studies. Moreover, although other LNAAs compete for transport across the blood-brain barrier [37], we still observed clear dose-dependent tyrosine effects on (dopamine- instead of e.g., serotonin-related) cognitive functioning, indicating a successful intervention. Of the total amount of LNAAs provided by the yoghurt and the supplemental dose of tyrosine, the largest amount was also tyrosine, i.e., 94.7% versus 0.9% tryptophan and 4.3% phenylalanine in for example the 100 mg/kg body weight dose of tyrosine with 140 mL yoghurt; the amount supplied to an average 70 kg individual. Nevertheless, future studies should also take blood plasma concentrations of the other LNAAs into account and calculate the plasma tyrosine/competing amino acids ratio to determine the selectivity of the intervention. Furthermore, additional assessment of plasma tyrosine concentrations at earlier time points, e.g., after 30 and/or 60 min after tyrosine ingestion, would be interesting to detect the exact moment where tyrosine concentrations peak in order to determine age-related differences in absorption and metabolic response.

In addition to increased baseline tyrosine levels, we observed higher (baseline-corrected) tyrosine plasma levels after the same dose in older versus young adults. This effect might be due to an age-related reduced first pass effect in the liver [3], such that higher amounts of tyrosine enter the blood stream in older adults. Moreover, age-related insulin resistance [35], or other kinetic effects [3,4,5] may contribute to reduced peripheral amino acid uptake from the blood.

On a cognitive level, relative to the lowest dose, higher doses of tyrosine were accompanied by negative effects on working memory performance in older adults in the most difficult N-back level. This adverse effect of tyrosine was linked to the peripheral plasma tyrosine responses, as greater dose-dependent increases in plasma tyrosine concentrations predicted greater cognitive detriments with increasing dose. Other studies using the same 150 mg/kg body weight tyrosine dose in young adults, which gave adverse effects versus the 100 mg/kg body weight dose in our older adults, have shown beneficial effects of tyrosine on working memory performance (e.g., [32,38]). The currently-observed decrements in working memory performance with increasing tyrosine doses in older adults might be surprising in the light of previous theories suggesting against dose-dependent effects in young adults [17,39]. However, the older adults showed markedly increased plasma tyrosine levels compared with the same dose in young adults, and this increase was associated with apparently paradoxical cognitive effects. This suggests that—due to peripheral changes in the aging body—much more tyrosine has reached, and probably crossed the blood-brain barrier in older adults. This high central precursor availability of probable peripheral origin, may have increased central catecholamine synthesis, subsequently leading to inhibition of tyrosine hydroxylase (TH), the enzyme that converts tyrosine to L-dopa (i.e., the final precursor of dopamine) [40]. In rats, for example, 250 mg/kg of phenylalanine, the precursor of tyrosine, increased dopamine release, 500 mg/kg had no effect, and 1000 mg/kg reduced dopamine release, suggesting a TH inhibitory mechanism [41]. Detrimental effects of increasing tyrosine dose may be less surprising when considering findings of increased dopamine synthesis capacity in older adults [25,26]. We speculate that administrating extra precursor to a system with already high dopamine synthesis capacity may result in its inhibition. Reduction of dopamine synthesis by inhibiting TH may also occur further in the dopamine signaling cascade, when an excess of dopamine increases dopamine D2 autoreceptor binding [42]. The aging brain might be more sensitive to overshoots in auto-regulation, for example due to increased inflammatory markers, such as cytokines [43], which can alter TH availability and autoregulatory dopamine transporter expression [44].

We did not have a placebo-controlled design and, hence, cannot infer whether tyrosine actually improved or diminished working memory performance in older adults relative to baseline. However, with the current design and results we do show that increasing doses of tyrosine result in detrimental working memory performance, questioning the cognitive enhancing potential of tyrosine in healthy aging, at least with the currently used higher doses. Future placebo-controlled designs should further test the beneficial or unfavorable effects of tyrosine administration on cognitive functioning in older adults, also including lower doses <100 mg/kg body weight.

## 5. Conclusions

In this double-blind, randomized cross-over trial, we observed a clear dose-response in plasma tyrosine after three different doses of tyrosine in older adults. Moreover, our data demonstrated that plasma tyrosine concentrations were markedly increased in older compared with young adults. Importantly, a high dose-dependent plasma tyrosine response was related to decrements in working memory performance in older adults with higher tyrosine doses. This study shows that age-related increases in plasma tyrosine response are associated with adverse dose-dependent effects of tyrosine administration on cognition.

## Figures and Tables

**Table 1:** Characteristics at screening of 17 young and 17 older adults assigned into the study.

	Young Adults (*n* = 17)	Older Adults (*n* = 17)	*p*-Value
Age (years)	21.5 ± 2.8 ^a^ (18–30)	69.6 ± 2.9 (65–74)	<0.001
Sex, Male (%)	47	47	1
Education Low/Middle/High (%)	0/6/94 ^b^	0/29/71	0.076
Body weight (kg)	68.3 ± 9.8	73.0 ± 13.2	0.25
BMI (kg/m^2^)	21.5 ± 1.6	25.2 ± 2.7	<0.001
MMSE score	N.A.	28 (27–29)^c^	-
Adjusted NART score	94.8 ± 4.6	103.6 ± 5.5	<0.001

^a^ Mean ± SD (all such values); ^b^ Dutch Education system: Low, Primary education; Middle, Vocational or secondary education; High, Senior vocational/academic or tertiary education; ^c^ Median (IQR); Abbreviations: BMI, Body Mass Index; MMSE, Mini-Mental State Examination; N.A., not applicable; NART, Dutch version of the National Adult Reading Test.

**Table 2:** Hits, misses, correct rejections, false alarms, and reaction times on the N-back task for the different tyrosine doses per age group.

		Young Adults	Older Adults		
		150 mg/kg (*n* = 17)	100 mg/kg (*n* = 17)	150 mg/kg (*n* = 16)	200 mg/kg (*n* = 17)
**Hits (%)**	0-back	100 ± 0.0 ^a^	98.2 ± 0.9	99.1 ± 0.5	99.2 ± 0.8
	1-back	98.6 ± 0.6	96.7 ± 1.0	97.2 ± 0.9	94.7 ± 3.0
	2-back	90.0 ± 1.7	85.9 ± 2.5	87.1 ± 2.7	85.1 ± 3.0
	3-back	78.3 ± 3.1	75.2 ± 2.9	68.1 ± 3.3	66.6 ± 4.0
**Misses (%)**	0-back	± 0.0	1.8 ± 0.8	0.9 ± 0.5	0.8 ± 0.8
	1-back	1.4 ± 0.5	3.3 ± 1.0	2.8 ± 0.9	5.7 ± 3.0
	2-back	10.0 ± 1.7	14.1 ± 2.5	12.9 ± 2.7	14.9 ± 3.0
	3-back	45.0 ± 21.8	24.8 ± 2.9	31.9 ± 3.3	33.4 ± 4.0
**Correct rejections (%)**	0-back	99.7 ± 0.1	98.8 ± 0.3	97.8 ± 1.9	97.4 ± 2.1
	1-back	99.1 ± 0.2	98.1 ± 0.6	96.5 ± 2.2	97.1 ± 1.2
	2-back	98.1 ± 0.3	94.8 ± 1.0	94.7 ± 1.4	95.6 ± 1.0
	3-back	96.3 ± 0.8	91.8 ± 1.1	91.4 ± 1.6	90.1 ± 1.3
**False alarms (%)**	0-back	0.3 ± 0.1	1.2 ± 0.3	2.2 ± 1.9	2.6 ± 2.0
	1-back	0.9 ± 0.2	1.9 ± 0.6	3.5 ± 2.2	2.8 ± 1.2
	2-back	1.9 ± 0.3	5.2 ± 1.0	5.3 ± 1.4	4.4 ± 1.0
	3-back	3.7 ± 0.8	8.2 ± 1.1	8.6 ± 1.6	9.91 ± 1.3
**Reaction time target (ms)**	0-back	457.6 ± 21.4	474.3 ± 24.3	481.7 ± 23.8	483.7 ± 23.9
	1-back	545.0 ± 26.4	546.5 ± 26.1	549.0 ± 23.2	548.2 ± 22.7
	2-back	629.8 ± 29.9	658.2 ± 32.4	645.1 ± 30.6	620.2 ± 27.8
	3-back	729.3 ± 45.3	738.1 ± 32.6	706.8 ± 35.1	713.5 ± 33.4

^a^ Values are mean ± SEM (Standard Error of the Mean).
